# Crystal Structures of Human Pyridoxal Kinase in Complex with the Neurotoxins, Ginkgotoxin and Theophylline: Insights into Pyridoxal Kinase Inhibition

**DOI:** 10.1371/journal.pone.0040954

**Published:** 2012-07-18

**Authors:** Amit K. Gandhi, Jigar V. Desai, Mohini S. Ghatge, Martino L. di Salvo, Stefano Di Biase, Richmond Danso-Danquah, Faik N. Musayev, Roberto Contestabile, Verne Schirch, Martin K. Safo

**Affiliations:** 1 Department of Medicinal Chemistry, Institute for Structural Biology and Drug Discovery, Virginia Commonwealth University, Richmond, Virginia, United States of America; 2 Dipartimento di Scienze Biochimiche and Istituto Pasteur – Fondazione Cenci Bolognetti, Sapienza Università di Roma, Roma, Italy; Weizmann Institute of Science, Israel

## Abstract

Several drugs and natural compounds are known to be highly neurotoxic, triggering epileptic convulsions or seizures, and causing headaches, agitations, as well as other neuronal symptoms. The neurotoxic effects of some of these compounds, including theophylline and ginkgotoxin, have been traced to their inhibitory activity against human pyridoxal kinase (hPL kinase), resulting in deficiency of the active cofactor form of vitamin B_6_, pyridoxal 5′-phosphate (PLP). Pyridoxal (PL), an inactive form of vitamin B_6_ is converted to PLP by PL kinase. PLP is the B_6_ vitamer required as a cofactor for over 160 enzymatic activities essential in primary and secondary metabolism. We have performed structural and kinetic studies on hPL kinase with several potential inhibitors, including ginkgotoxin and theophylline. The structural studies show ginkgotoxin and theophylline bound at the substrate site, and are involved in similar protein interactions as the natural substrate, PL. Interestingly, the phosphorylated product of ginkgotoxin is also observed bound at the active site. This work provides insights into the molecular basis of hPL kinase inhibition and may provide a working hypothesis to quickly screen or identify neurotoxic drugs as potential hPL kinase inhibitors. Such adverse effects may be prevented by administration of an appropriate form of vitamin B_6_, or provide clues of how to modify these drugs to help reduce their hPL kinase inhibitory effects.

## Introduction

Some well known drugs that are directed at different targets have also been shown to inhibit human pyridoxal kinase (hPL kinase) activity with a concomitant deficiency in pyridoxal 5′-phosphate (PLP) causing unwanted neurotoxic side effects, such as peripheral neuropathy, unconsciousness, convulsions or seizures, sleeplessness, headache, restlessness, agitation, tremors, and hallucination [1]–[7]. Vitamin B_6_ in its active form, namely PLP, is a cofactor for over 160 enzymatic activities (PLP-dependent enzymes) serving vital roles in neurotransmitter production, as well as in several other essential pathways [8]. For example, PLP-dependent enzymes are involved in the biosynthesis of D-serine, D-aspartate, L-glutamate, glycine, γ-aminobutyric acid (GABA), serotonin, epinephrine, norepinephrine, histamine and dopamine. A decrease in GABA level, induced by antivitamin B_6_ agents, is known to be accompanied by epileptic seizures [9]. Several of these agents, such as progabide, theophylline, and ginkgotoxin are potent hPL kinase inhibitors [1]–[5], [10]–[21], resulting in PLP deficiency with a concomitant reduction in PLP-dependent enzyme activities, such as that of glutamate decarboxylase, which catalyzes formation of GABA from L-glutamate. It has long been recognized that co-administration of pyridoxine, the primary dietary form of vitamin B_6_ together with these hPL kinase inhibitors reduce or prevent their associated neurotoxic side effects [5], [17], [22], [23].

**Figure 1:**
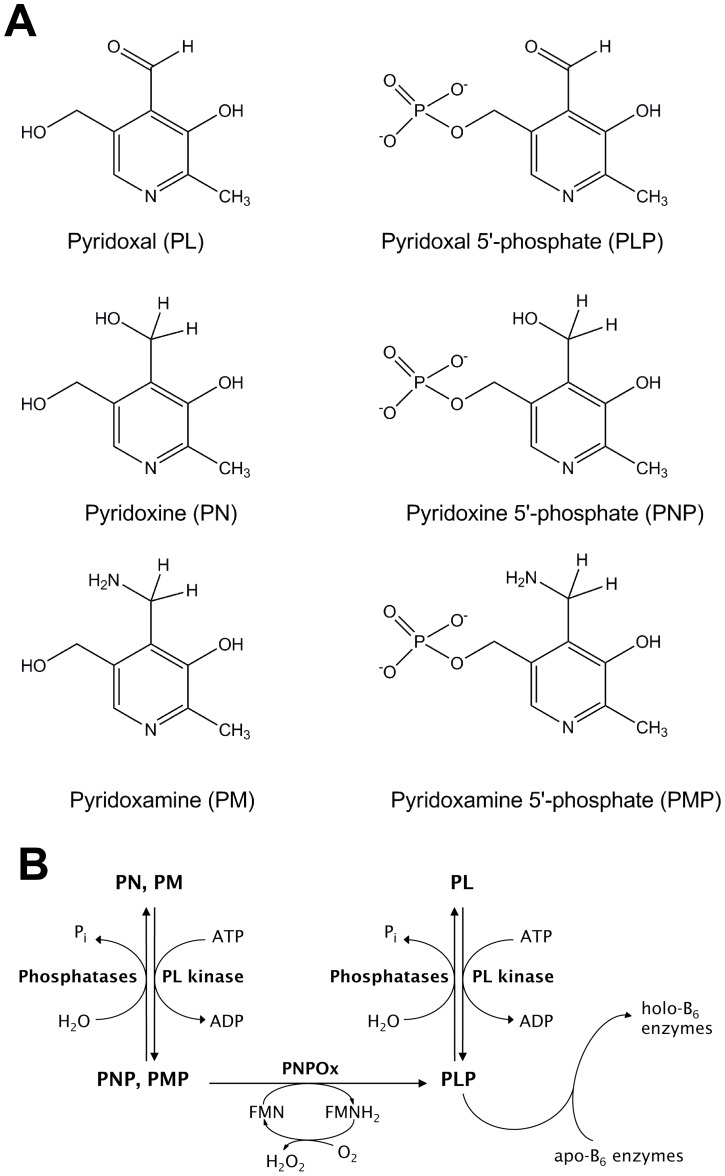
(A) Structures of B_6_ vitamers. (B) Reactions in vitamin B_6_ metabolism: scheme of the interconversion of B_6_ vitamers by PL kinase, pyridoxine 5′-phosphate oxidase and different phosphatases.

PL kinase is one of the key enzymes involved in PLP metabolism [24]. In the presence of MgATP, this enzyme catalyzes the phosphorylation of the three inactive primary forms of vitamin B_6_, i.e. pyridoxine (PN), pyridoxamine (PM), and pyridoxal (PL) to their 5′-phosphorylated forms, PNP, PMP and PLP, respectively (Fig. 1A and B). PNP and PMP are subsequently converted to PLP (Fig. 1B) by pyridoxine 5′-phosphate oxidase (PNPOx) [24]. During the turnover of PLP-dependent enzymes, PLP is released and converted back to PL (Fig. 1B) by different phosphatases, and subsequently re-phosphorylated to PLP (Fig. 1B) by PL kinase [24]–[26]. The structure of PL kinase has been determined from several sources [27]–[32]. PL kinase is a homodimer with each active site exclusively formed by a single monomer. The ATP binds in a shallow cavity at the active site, while the vitamin B_6_ substrate binds in a solvent-inaccessible deeper cavity opposite but facing the γ-phosphate of the ATP.

**Figure 2:**
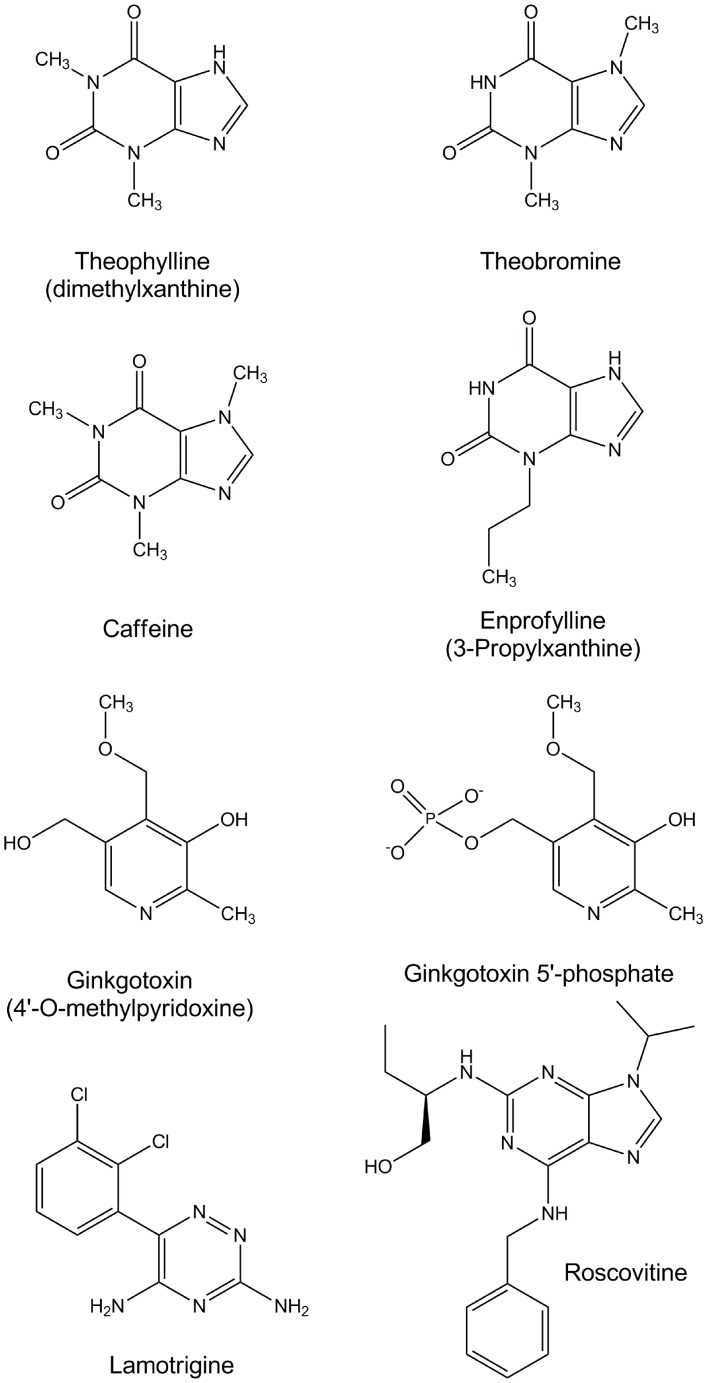
Structures of potential PL kinase inhibitors.

Theophylline (Fig. 2) is a xanthine drug used in therapy for respiratory diseases, e.g. chronic obstructive pulmonary disease or asthma. Theophylline has been shown to significantly decrease plasma PLP levels in animals, asthmatic patients, and healthy volunteers, resulting in the above described neurotoxicity [16], [18], [23]. A plasma concentration of theophylline higher than 110 µM is known to be associated with these symptoms [16]. Theophylline is also naturally found in trace amount in tea, and as much as 3.7 mg/g in certain types of cocoa beans [33]. Several other xanthines, including theobromine, enprofylline and caffeine (Fig. 2) also occur naturally in coffee and cocoa and have also been used as bronchodilators for treating asthma and/or as stimulants [33]–[35]. Similar to theophylline, these compounds are known to exhibit neurotoxic effects [33], [35]–[37], although it is not clear whether these side effects are related to hPL kinase inhibition or PLP deficiency in the cell.

Ginkgotoxin (4′-O-methylpyridoxine, an analog of vitamin B_6_) (Fig. 2), found in *Ginkgo biloba* seeds also leads to significant PLP deficiency in mammals, resulting in neuronal symptoms similar to those of theophylline [7], [38], [39]. Various medications from *Ginkgo biloba* are easily available over the counter and are widely used in the treatment of several conditions ranging from bronchial asthma, irritable bladder, depression, dizziness, tinnitus and several others [1], [7], [22], [38]. These medications have a prominent presence in traditional Chinese and Japanese medicine, and in recent times also in European medicine. Food poisoning, and in some cases death(both in humans and cattle) has been reported in Japan and South Africa due to ginkgotoxin [22], [40].

We have determined the crystal structures of hPL kinase in complex with ginkgotoxin and theophylline to gain molecular insights into the inhibitory activities of these compounds. We also report kinetic studies on other compounds or drugs that show structural similarity to theophylline and/or exhibit neurotoxic effects, including enprofylline, theobromine, caffeine, and lamotrigine (Fig. 2) to determine whether these compounds could inhibit hPL kinase and potentially lead to depletion of PLP in the cell. This study could serve as a guide to identify or recognize neurotoxic drugs as potential hPL kinase inhibitors and thus may offer a rational for pharmacological intervention.

## Results

### Human PL Kinase Inhibitors

We tested the inhibitory activities of ginkgotoxin and theophylline on hPL kinase, as well as that of other xanthines including enprofylline, theobromine and caffeine, due to their structural similarities with theophylline. We also tested the inhibitory activity of the anticonvulsant lamotrigine, another neurotoxic drug. At 100 µM concentration, ginkgotoxin, theophylline, lamotrigine, enprofylline, theobromine, and caffeine inhibited hPL kinase activity by 100%, 60%, 45%, 33%, 22% and 21%, respectively. Detailed kinetic studies with ginkgotoxin, theophylline, lamotrigine and enprofylline showed the compounds to inhibit hPL kinase with a K_i_ of 3, 50, 56 and 228 µM, repectively. These kinetic results correlate well with other studies that reported a drop in serum concentration of PLP of normal or epileptic patients when treated with theophylline or lamotrigine [16], [41].

Previous kinetic studies by Lainé-Cessac *et al.* [10] found theophylline to inhibit hPL kinase with K_i_ of 3 µM, which is significantly lower than our experimental value of 50 µM. The investigators used unpurified protein (erythrocyte preparation) and their assay was based on HPLC separation and fluorometric detection of PL and PLP, as opposed to the direct continuous spectrophotometric assay used in our studies. The investigators reported a significantly lower K_m_ of 1 µM for PL compared to the ∼60 µM reported in the literature, and also observed in our current studies (K_m_ of 58 µM) with purified protein. Our study showed ginkgotoxin to inhibit hPL kinase with a K_i_ of 3 µM which is different from the previously reported value of 0.414 µM. The K_m_ values for PL reported by these investigators and by us are similar [1]. The same investigators reported that hPL kinase was able to phosphorylate ginkgotoxin [1].

### Structure of the Binary hPL Kinase-theophylline Complex

**Figure 3:**
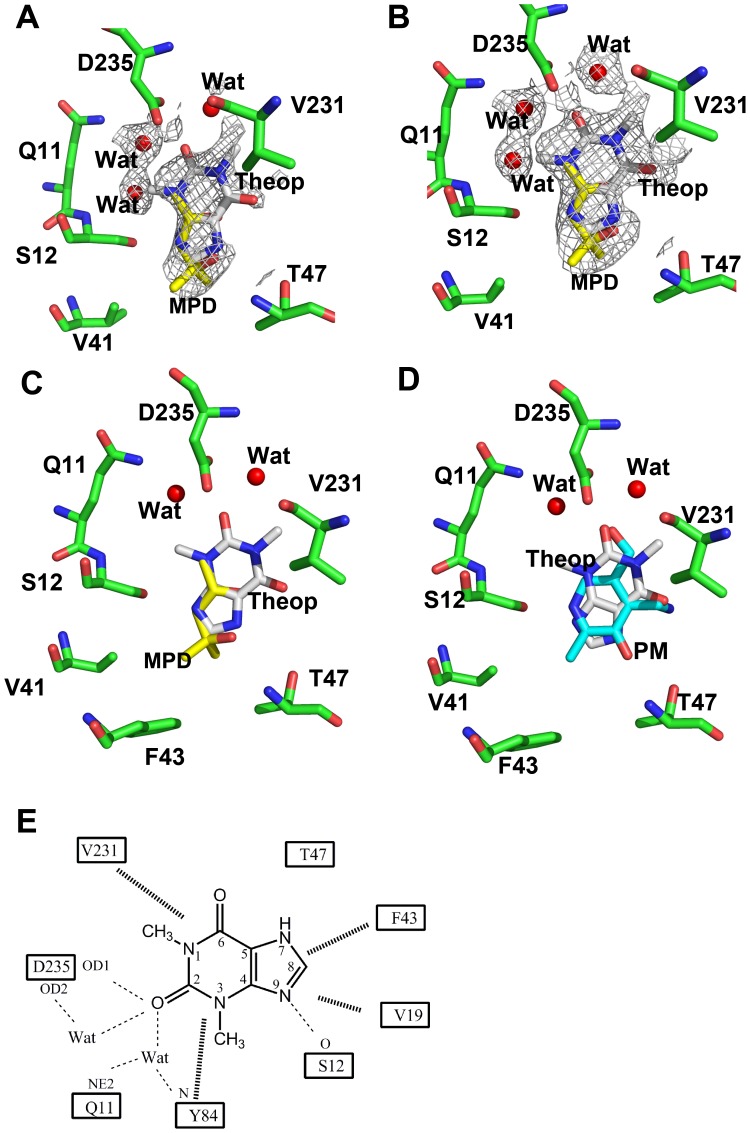
Binding of theophylline at the active site of hPL kinase. (A) A Fo-Fc map (contoured at 2.5 σ level) of MPD, theophylline and active site water molecules of the hPL kinase structure, calculated before the MPD, theophylline and water molecules were added to the refined model. (B) A 2Fo-Fc map (contoured at 0.9 σ level) of MPD, theophylline and water molecules of the hPL kinase structure. Both maps are superimposed with the final refined models. (C) Interactions between the active site residues (green sticks) and theophylline (white sticks) and MPD (yellow sticks). Water molecules are red sphere. (D) Superimposed binding of theophylline (from hPL kinase) and pyridoxamine (from sheep PL kinase). Protein residues are green stick from the hPL kinase, theophylline is white stick, pyridoxamine is cyan stick, and water molecules are red sphere. (E) Schematic diagram showing interactions between the active site residues, water molecules and theophylline. Dotted and heavy lines are hydrogen-bond and hydrophobic interactions, respectively. Only potential hydrogen-bond interactions less than 3.6 Å are shown with dotted lines. For brevity, theophylline is denoted as theop in the figure.

Human PL kinase was co-crystallized with theophylline and refined to 2.1 Å resolution, using the isomorphous hPL kinase D235A mutant structure (PDB code 3FHX). The electron densities for all main-chain residues are clearly interpretable with the exception of the first three residues from the N-terminus, residues 208–213 and 280–281 from the A and B-subunits, respectively. The overall dimeric structure is very similar to the previously published unliganded hPL kinase wild-type structure (PDB code 2YXU). Theophylline is bound at the PL binding site in both subunits, with an occupancy of 60% (Fig. 3A–C). Occupying the same position as the theophylline is a 2-methyl-2,4-pentanediol (MPD) molecule at 40% occupancy (Fig. 3A–C). Our previously reported unliganded wild-type human PL kinase structure also showed MPD fully bound at the active site [32]. Figure 3D shows a structural comparison between the bound theophylline in hPL kinase and bound pyridoxamine (PM) in a previously published sheep PL kinase structure [28]. The two ligands superimpose closely, with several conserved protein interactions. Possible contacts between the active site residues and theophylline (Fig. 3C and E) involve a hydrogen-bond interaction between the hydroxyl group of Ser12 and the N9 of the theophylline imidazole nitrogen, which is also present between Ser12 and the pyridine nitrogen atom of PM in the sheep PL kinase structure. In the sheep structure, there are two hydrogen-bond interactions formed between the hydroxyl and amide nitrogen of Thr47 with the hydroxyl group of PM. In the theophylline complex, the C6 oxygen of the piperazine ring and the N7 of the imidazole ring are adjacent to Thr47, but any possible hydrogen-bond interactions are considerably lengthened beyond 3.6 Å. In our previous studies we have shown that Asp235 in PL kinase acts as the active site base to deprotonate the C5′-OH group of B_6_ vitamers prior to phosphorylation [42]. In the sheep structure, Asp235 makes a strong hydrogen-bond interaction with the C5′-OH group of PM. This interaction is conserved with the C2 oxygen of theophylline. In addition the C2 oxygen atom is involved in water-mediated interactions with the side-chains of Asp235, Gln11 or main-chain nitrogen of Tyr84. Both PM and theophylline make hydrophobic interactions with the protein residues Val231, Val19, Tyr84 and Phe43. We should point out that, it’s quite possible that theophylline binds in different alternate conformations; however, such conformers would abolish several of the hydrogen-bond interactions described above for the modeled conformer. As previously described in the wild-type unliganded hPL kinase structure, the bound MPD at the active site makes a hydrogen bond interaction with the main-chain nitrogen atom of Thr47, as well as several hydrophobic interactions with the active site residues. Also, like the previously published wild-type unliganded hPL kinase structure, we observed several MPD molecules located on the surface of the protein at various crevices, as well as at the interfaces of crystal contacts. MPD was used as an additive during crystallization.

A bound Na^+^ in the hPL kinase-theophylline structure is located at the ATP binding site, and is coordinated by five well-defined water molecules. In the previous unliganded hPL kinase structure we also showed a similarly bound Na^+^. The intricate water-mediated interaction between the Na^+^ and the protein is believed to stabilize the active site conformation [32]. It was shown that binding of MgATP displaces the Na^+^ to another position, where it makes mediated interactions between the ATP γ-phosphate and the protein residues, contributing to the stabilization of the nucleotide [32].

### Structure of the Ternary hPL Kinase-ginkgotoxin-MgATP Complex

**Figure 4:**
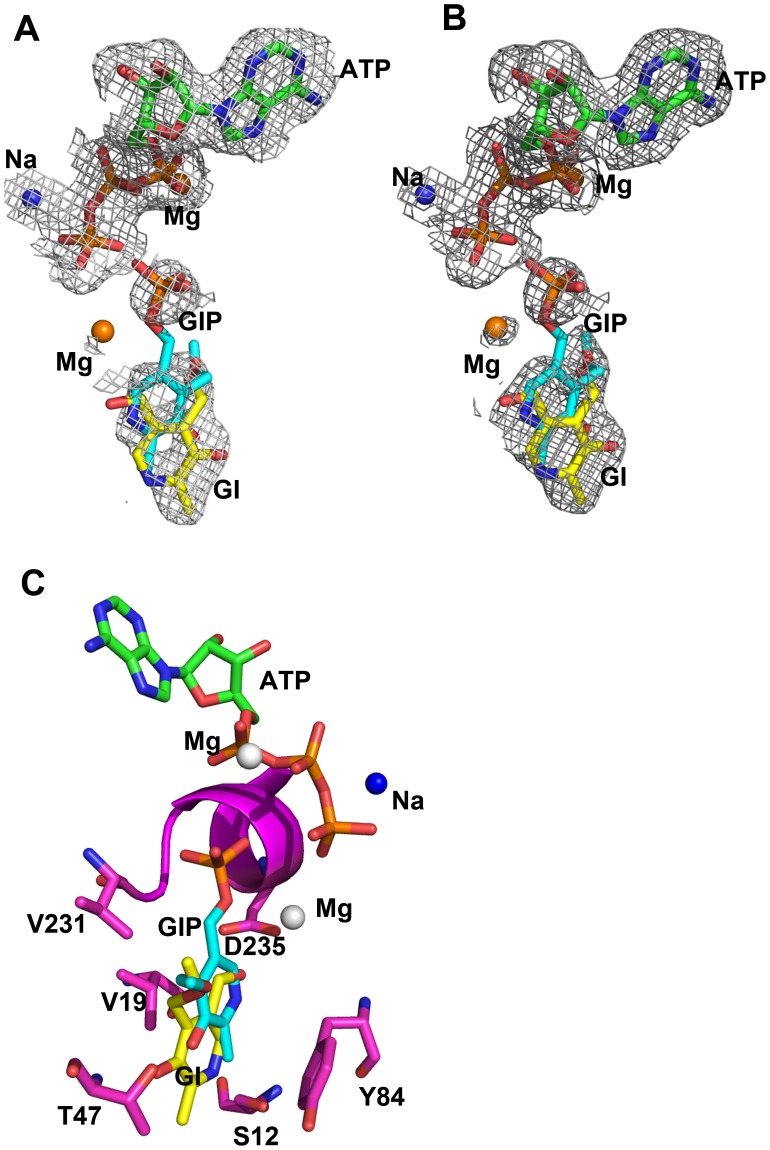
Binding of ginkgotoxin and its phosphorylated analog at the active site of hPL kinase. (A) A Fo-Fc map (contoured at 2.5 σ level) of ginkgotoxin and its phosphorylated analog, ATP, Mg^2+^ and Na^+^ of the hPL kinase structure, calculated before these molecules were added to the refined model. (B) A 2Fo-Fc map (contoured at 0.9 σ level) of ginkgotoxin and its phosphorylated analog, ATP, Mg^2+^ and Na^+^ of the hPL kinase structure. Both maps are superimposed with the final refined models. (C) Binding of ginkgotoxin (yellow stick), phosphorylated ginkgotoxin (cyan stick), ATP (green and brown sticks), Mg^2+^ (white sphere) and Na^+^ (blue sphere). Protein residues are in magenta stick or ribbon. For brevity, ginkgotoxin and its phosphorylated analog are denoted as GI and GIP in the figure.

We also co-crystallized hPL kinase with ginkgotoxin (4′-O-methylpyridoxine) and MgATP, and the ternary complex structure was refined to 2.15 Å resolution. The first three N-terminus residues had weak density and were not included in the final refined model. Also, the overall structure is indistinguishable from the wild-type unliganded structure. We observed bound MgATP and ginkgotoxin (Fig. 4A–C) at both subunit active sites. The binding mode of MgATP is conserved as previously described for the wild-type hPL kinase-MgATP complex [32]. The ATP adenine moiety makes both hydrogen-bond and hydrophobic contacts with the protein. The three ATP phosphate groups are involved in extensive hydrogen-bond interactions with the protein, including a P-loop consisting of an anion hole formed by the highly conserved sequence motif GTGA (residues 232–235) and the N-terminus of a α7-helix formed by residues 234–248. The ATP β- and γ-phosphates are further stabilized by bound Mg^2+^ and Na^+^, the former ion being associated with both phosphate groups, while the Na^+^ is only associated with the γ-phosphate. These metals help to neutralize the negative phosphate and the active site acidic residue charges and stabilize the transition state during the γ-phosphate transfer from ATP to the substrate [32].

**Figure 5:**
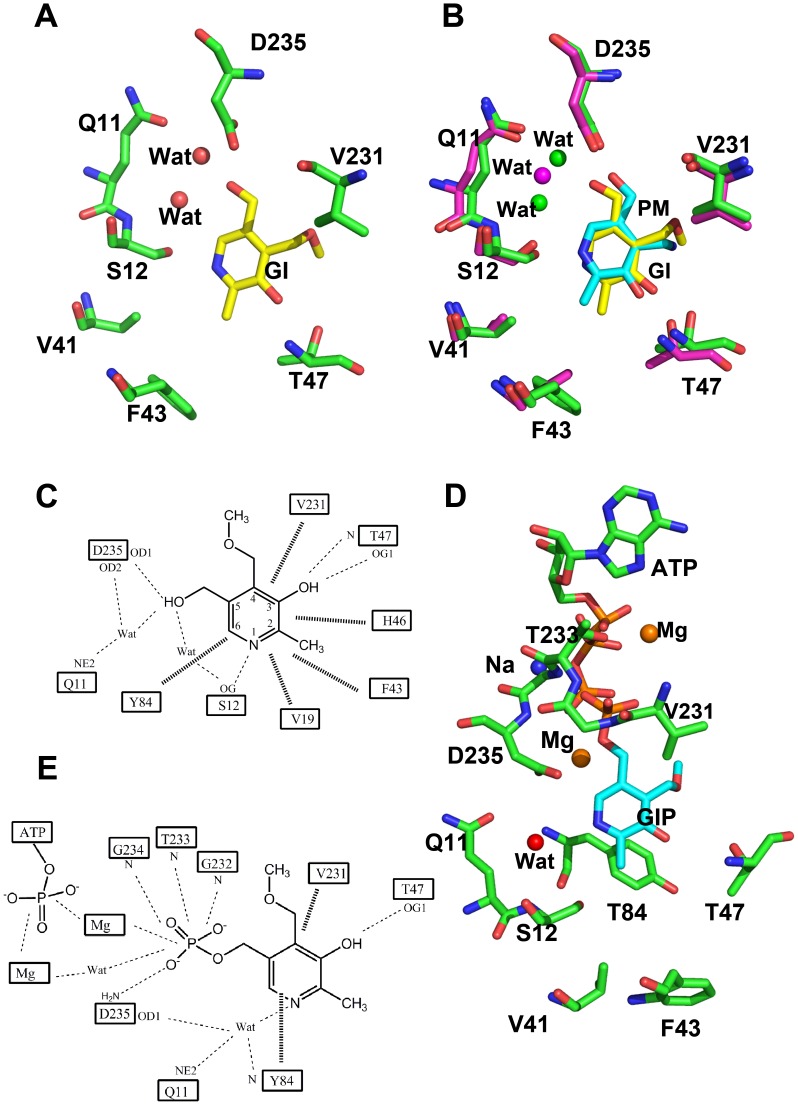
(A) Interactions between ginkgotoxin (yellow sticks) and the active site residues (green stick). Water molecules are red sphere. (B) Superimposed binding of ginkgotoxin (yellow stick, from hPL kinase) and pyridoxamine (cyan stick from sheep PL kinase). Protein residues are green and cyan sticks for the hPL kinase and sheep PL kinase, respectively. Water molecules are green and red spheres for the hPL kinase and sheep PL kinase, respectively. (C) Schematic diagram showing interactions between active side residues, water molecules and ginkgotoxin. Dotted and heavy lines are hydrogen-bond and hydrophobic interactions, respectively. (D) Interactions between active site residues (green stick), ginkgotoxin phosphate (cyan stick), ATP (green and brown sticks), Mg ions (brown sphere), Na ions (blue sphere) and water molecules (red sphere). (E) Schematic diagram showing interactions between ginkgotoxin phosphate, ATP, water molecules, Mg ions and the protein residues. Dotted and heavy lines are hydrogen-bond and hydrophobic interactions, respectively. Only potential hydrogen-bond interactions less than 3.6 Å are shown with dotted lines. For brevity, ginkgotoxin and its phosphorylated analog are denoted as GI and GIP in the figure.

The two molecules of ginkgotoxin are bound to the PL binding sites of the two enzyme monomers (Fig. 4A–C). We also observed what appears to be the phosphorylated product of ginkgotoxin bound at the PL sites, overlapping the bound unphosphorylated ginkgotoxin, although the electron density map showed a break in the ring structure and the phosphate moiety (Fig. 4A and B). The ginkgotoxin phosphate position has previously been observed to bind sulfate and/or phosphate in PL kinase, making similar interactions with the anion hole of the kinase [43]. Ginkgotoxin and its phosphorylated analog were refined with occupancies of 60% and 40%, respectively. We have previously reported in the hPL kinase D235A mutant structure, a similar co-existence of PL and PLP at the active site [42]. The interactions between ginkgotoxin and the protein (Fig. 5A–C) are identical to those previously described for PM in the sheep PL kinase structure. These include hydrogen-bond interactions from N1 to the hydroxyl group of Ser12; C5′-OH group to the carboxylate of Asp235; C3 oxygen to both the amide nitrogen and hydroxyl group of Thr47. There are also hydrophobic interactions between ginkgotoxin and Thr47, Phe43, Val231, Val19, His46 and Tyr84 that are conserved in the sheep structure. In particular, the methyl ether group makes hydrophobic interactions with Thr47 and Val231 that could contribute to the binding of ginkgotoxin to PL kinase. There appears to be a network of water-mediated hydrogen-bond interactions involving two water molecules and the C5′-OH group of ginkgotoxin, and the main-chain nitrogen of Ser12, the side chains of Gln11 and Asp235. Only one water molecule appears at the sheep active site, and the water-mediated interactions are either missing or significantly lengthened in the sheep PL kinase structure complexed with PM. These additional water-mediated interactions could also be contributing to the potent inhibitory activity of ginkgotoxin.

The pyridine ring of the phosphorylated ginkgotoxin is displaced about 2 Å from that of the unphosphorylated analog in the direction of the bound ATP, resulting in extinction or significant lengthening of the hydrogen-bond interaction from N1 to Ser12 and C3 oxygen to Thr47 (Figs. 4C, 5D and 5E). The N1 atom makes a water-mediated hydrogen-bond interaction with the side-chain atoms of Gln11, Asp235 or main-chain nitrogen atom of Tyr84. The displacement of the ginkgotoxin phosphorylated analog toward ATP has placed its phosphate group about 3 Å from the ATP γ-phosphate group, as compared to the ∼6 Å closest distance between the ginkgotoxin C5′-OH and the ATP γ-phosphate. Like the hPL kinase D235A structure [42], we also observed a second bound Mg^2+^ at each active site that lies close to the ginkgotoxin phosphate and mediates an interaction with the ATP γ-phosphate, helping to dissipate the negative charges. This second Mg^2+^ does not occur in structures lacking a bound phosphorylated compound. The α- and β-phosphate groups are also stabilized by extensive interactions with the P-loop residues as described above. Finally, the ATP γ-phosphate is further stabilized by the conserved Na^+^ and Mg^2+^.

## Discussion

Several medicinal compounds are known to exhibit neurotoxic effects, which have been traced to their inhibitory activity against human PL kinase with concomitant PLP deficiency [1]–[7]. Two such potent reported hPL kinase inhibitors are theophylline and ginkgotoxin [1]–[5], [10]–[21]. To gain insight into how these compounds affect vitamin B_6_ metabolism and the concomitant PLP deficiency, we performed structural studies of hPL kinase co-crystallized with these compounds. Both compounds bind at the PL binding site, which might explain their inhibitory properties against PL kinase. Kaster *et al.* showed that ginkgotoxin competes with PL and that, in the presence of ginkgotoxin; phosphorylation of PL is severely hindered [1]. Nevertheless, they also observed that when PL concentration is increased the inhibitory effect of ginkgotoxin or theophylline is alleviated; consistent with several studies showing that poisoning from ginkgotoxin and theophylline can be reversed by vitamin B_6_ [5], [17], [22], [23]. It is a common practice to co-administer vitamin B_6_ with drugs that are suspected to lead to PLP deficiency.

We have also identified several other potential hPL kinase inhibitors, including enprofylline, theobromine, caffeine and lamotrigine using kinetic studies. These compounds, like ginkgotoxin and theophylline, also exhibit neurotoxic effects. Moreover, a previous study on epileptic patients treated with lamotrigine, showed, as for theophylline, a drop in the serum concentration of PLP [41].

It is interesting to note that not only do the tested compounds fit in the PL binding site, but they also have similarly placed heteroatoms that can potentially make conserved interactions with the active site residue. We note that unlike theophylline, the N7 imidazole nitrogen of caffeine and theobromine (Fig. 2) are methylated and may not be available to make hydrogen-bond interactions with the Ser12 hydroxyl group as observed in theophylline. This may explain the lower PL kinase inhibitory activities exerted by these two compounds. Although not obvious, it seems that the reduced kinase inhibitory activity by enprofylline compared to theophylline could be due to steric crowding by the enprofylline propyl moiety (Fig. 2). Roscovitine, an inhibitor of cyclin-dependent kinases with strikingly similar core structural features as theophylline (Fig. 2) has also been shown to have moderate inhibitory activity against hPL kinase [44], [45]. Structural studies show this compound to bind to hPL kinase at the PL site making conserved protein interactions in a similar fashion as theophylline [45]. It thus seems that the active site of hPL kinase is a sink for compounds with uniquely placed moieties that are capable of making interactions with the active site. If compounds with such structural characteristics are known to be neurotoxic, they should be carefully investigated to find whether they affect B_6_ metabolism by inhibiting hPL kinase. A co-administration of vitamin B_6_ can be recommended with their therapeutic use.

## Materials and Methods

### Materials

Ginkgotoxin was synthesized by 4′-O-methylation of pyridoxine according to published method [39]. Theophylline, enprofylline, theobromine, caffeine, and lamotrigine were purchased form Sigma-Aldrich (St. Louis, MO) and used without further purification. Wild-type hPL kinase used for the kinetic and crystallization experiments was expressed and purified as previously published by our group [32].

### Determination of Kinetic Constants

Wild-type hPL kinase used in the kinetic experiments was dialyzed overnight against 20 mM sodium BES buffer, pH 7.2. All assays were performed at 37°C in a 1-cm thermostated cuvette. Initial velocity studies for the conversion of PL to PLP were followed at 388 nm in an Agilent 8454 UV/Vis diode array spectrophotometer in 20 mM sodium BES buffer, pH 7.2 [32]. As a first step, percentage inhibition of hPL kinase with each tested drug (100 µM) was measured at a saturating MgATP concentration of 1 mM and fixed PL concentration of 200 µM (approximately three times the K_m_ value). Detailed kinetic studies for the determination of K_i_ for theophylline, enprofylline, lamotrigine and ginkgotoxin were carried out at several different drug concentrations (e.g. 10, 25, 50, 75, 100 and 150 µM) under two experimental conditions: at a MgATP concentrations of 800 µM and varied PL concentrations between 2 and 340 µM, and reciprocally at a PL concentrations of 100 µM and varied MgATP concentrations between 50 µM and 2 mM. The reciprocal rate data were plotted against reciprocal of PL concentrations to obtain Lineweaver-Burk plots.

### Crystallization, Data Collection and Processing

Human PL kinasewas dialyzed overnight against 20 mM sodium BES buffer, pH 7.2 containing 150 mM NaCl and 5 mM 2-mercaptoethanol, and then concentrated to 25–35 mg/ml. Crystallization attempts were focused on previously published human PL kinase crystallization condition [32]. X-ray quality crystals with the hanging-drop method using PL kinase (700 µM) with theophylline (2.5 mM) or PL kinase with ginkgotoxin (2 mM) and MgATP (1 mM) and the precipitant 48–50% MPD at room temperature were obtained for the binary PL kinase-theophylline complex and the ternary PL kinase-ginkgotoxin-MgATP complex.

Crystals of the theophylline complex were cryo-protected in solution containing mother-liquor solution, 2.5 mM theophylline and 50% MPD; while crystals of the ginkgotoxin complex were cryo-protected in mother-liquor solution containing 1 mM MgATP, 2 mM ginkgotoxin and 50% MPD prior to data collection. X-ray data were collected at 100°K using a Rigaku X-Stream Cryogenic Crystal Cooler System and an R-Axis IV++ image plate detector, a Rigaku MicroMax–007 X-ray source equipped with Rigaku Varimax confocal optics operating at 40 kV and 20 mA. The data were processed with the Rigaku d*trek software and the CCP4 suite of programs [46]. The X-ray data are summarized in Table 1.

**Table 1:** Refinement parameters for the human PL kinase structure with bound inhibitors.

	Theophylline	Ginkgotoxin
**Data Collection Statistics**		
**Space Group**	I222	I222
**Cell Dimensions (Å)**	92.30, 115.85, 171.90	92.74, 115.22, 169.55
**Resolution (Å)**	37.68–2.10 (2.18–2.10)	28.82–2.15 (2.23–2.15)
**No. of measurements**	157598	212374
**Unique reflections**	52989 (5317)	49008 (4907)
**<I/sigma I>**	18.5 (4.1)	11.9 (4.1)
**Completeness (%)**	98.1 (98.0)	98.6 (100)
**Rmerge (%)** b	3.2 (24.1)	6.5 (33.5)
**Structure Refinement**		
**Resolution limit (Å)**	29.19–2.10 (2.18–2.10)	28.81–2.15 (2.23–2.15)
**No. of reflections**	52986 (5205)	45953 (4283)
**Rfactor (%)**	20.5 (33.7)	21.9 (42.5)
**Rfree (%)** c	25.4 (37.2)	26.2 (42.5)
**Rmsd standard geometry**		
**Bond-lengths (Å)**	0.006	0.010
**Bond-angles (°)**	1.30	1.60
**Dihedral angles**		
**Most favored regions**	90.3	89.9
**Additional allowed regions**	9.5	9.9
**Bfactors**		
**All atoms**	48.1	42.5
**Protein alone**	46.8	41.9
**Theophylline/Ginkgotoxin**	64.4	60.2
**Metal ions**	84.1	33.3
**Sulfate**	64.4	58.1
**Water**	56.2	43.3
**MPD**	71.6	67.3
**ATP**	–	34.8

a Numbers in parenthesis refer to the outermost resolution bin.

b Rmerge  =  Σ*_hkl_*Σ*_i_*|*I_hkli_* - 〈*I_hkli_*〉|/Σ*_hkl_*Σ*_i_*〈*I_hkli_*〉.

c 5% of the reflection were excluded from the refinement and used for the calculation of Rfree.

### Structure Refinement of the Binary PL Kinase-theophylline Complex

The isomorphous hPL kinase D235A mutant (PDB code 3FHX) structure stripped of all small molecule ligands, water and metals was used as the starting model for the refinement of the theophylline bound structure. All refinements were performed with the CNS program [47]. After rigid body refinement, and subsequent conjugate gradient minimization, simulated annealing and B-factor refinements, theophylline density was identified at the two PL binding sites of both subunits. Densities were also identified for the Asp235 side chains, as well as Na^+^ in both subunit active sites. These molecules were added, and the ensuing model subsequently refined with alternate cycles of conjugate gradient minimization, simulated annealing and B-factor refinements with intermittent model rebuilding and structure validation with COOT [48]. Addition of 337 water, 12 MPD and 7 sulfate molecules led to the final crystallographic Rfree and Rfactors of 20.5/25.4% at 2.1 Å resolution.

### Structure Refinement of the Ternary PL Kinase-ginkgotoxin-MgATP Complex

The above refined hPL kinase-theophylline complex structure was used as the starting model for structure refinement, following similar procedures as described above. We observed a bound ginkgotoxin and what appears to be the phosphorylated product of ginkgotoxin at both active sites. One ATP molecule, one Na ion, and two Mg ions were also identified in each of the two active sites. The final refined model at Rfree/Rfactors of 21.9/26.2% at 2.15 Å resolution contained 2 ginkgotoxin, 2 ATP, 273 water, 5 sulfate and 6 MPD molecules.

The structure solution/refinement statistics for the two complexes are shown in Table 1. All figures were drawn using PyMOL (Delano Scientific, 2006; http://www.pymol.org) and labels were added using Adobe® Photoshop.

### Data Deposition

The atomic coordinates and structure factors have been deposited in the RCSB Protein Data Bank with accession codes 4EOH and 4EN4 for the theophylline-bound and ginkgotoxin-bound hPL kinase structures, respectively.

## References

[1] Kastner U, Hallmen C, Wiese M, Leistner E, Drewke C (2007). The human pyridoxal kinase, a plausible target for ginkgotoxin from ginkgo biloba.. FEBS J.

[2] Seto T, Inada H, Kobayashi N, Tada H, Furukawa K (2000). Depression of serum pyridoxal levels in theophylline-related seizures.. No To Hattatsu.

[3] Kuwahara H, Noguchi Y, Inaba A, Mizusawa H (2008). Case of an 81-year-old woman with theophylline-associated seizures followed by partial seizures due to vitamin B_6_ deficiency.. Rinsho Shinkeigaku.

[4] Bonner AB, Peterson SL, Weir MR (1999). Seizures induced by theophylline and isoniazid in mice.. Vet Hum Toxicol.

[5] Steichen O, Martinez-Almoyna L, De Broucker T (2006). Isoniazid induced neuropathy: Consider prevention.. Rev Mal Respir.

[6] Spinneker A, Sola R, Lemmen V, Castillo MJ, Pietrzik K (2007). Vitamin B_6_ status, deficiency and its consequences-an overview.. Nutr Hosp.

[7] Wada K (2005). Ginkgo seed food poisoning. Chudoku Kenkyu..

[8] Eliot AC, Kirsch JF (2004). Pyridoxal phosphate enzymes: Mechanistic, structural, and evolutionary considerations.. Annu Rev Biochem.

[9] Nitsch C, Okada Y (1976). Differential decrease of GABA in the substantia nigra and other discrete regions of the rabbit brain during the preictal period of methoxypyridoxine-induced seizures.. Brain Res.

[10] Laine-Cessac P, Cailleux A, Allain P (1997). Mechanisms of the inhibition of human erythrocyte pyridoxal kinase by drugs.. Biochem Pharmacol.

[11] Delport R, Ubbink JB, Vermaak WJ, Becker PJ (1993). Theophylline increases pyridoxal kinase activity independently from vitamin B6 nutritional status.. Res Commun Chem Pathol Pharmacol.

[12] Delport R, Ubbink JB, Bosman H, Bissbort S, Vermaak WJ (1990). Altered vitamin B6 homeostasis during aminophylline infusion in the beagle dog.. Int J Vitam Nutr Res.

[13] Ubbink JB, Bissbort S, Vermaak WJ, Delport R (1990). Inhibition of pyridoxal kinase by methylxanthines.. Enzyme.

[14] Ubbink JB, Vermaak WJ, Delport R, Serfontein WJ, Bartel P (1990). The relationship between vitamin B6 metabolism, asthma, and theophylline therapy.. Ann NY Acad Sci.

[15] Ubbink JB, Delport R, Becker PJ, Bissbort S (1989). Evidence of a theophylline-induced vitamin B6 deficiency caused by noncompetitive inhibition of pyridoxal kinase.. J Lab Clin Med.

[16] Delport R, Ubbink JB, Serfontein WJ, Becker PJ, Walters L (1988). Vitamin B6 nutritional status in asthma: The effect of theophylline therapy on plasma pyridoxal-5′-phosphate and pyridoxal levels.. Int J Vitam Nutr Res.

[17] Bartel PR, Ubbink JB, Delport R, Lotz BP, Becker PJ (1994). Vitamin B-6 supplementation and theophylline-related effects in humans.. Am J Clin Nutr.

[18] Weir MR, Keniston RC, Enriquez JI, McNamee GA (1990). Depression of vitamin B6 levels due to theophylline.. Ann Allergy.

[19] Weir MR, Keniston RC, Enriquez JI, McNamee GA (1991). Depression of vitamin B6 levels due to dopamine.. Vet Hum Toxicol.

[20] Glenn GM, Krober MS, Kelly P, McCarty J, Weir M (1995). Pyridoxine as therapy in theophylline-induced seizures.. Vet Hum Toxicol.

[21] Alao AO, Yolles JC (1998). Isoniazid-induced psychosis. Ann. Pharmacother..

[22] Leistner E, Drewke C (2010). Ginkgo biloba and ginkgotoxin.. J Nat Prod 73, 86–92.

[23] Ubbink JB, Delport R, Bissbort S, Vermaak WJ, Becker PJ (1990). Relationship between vitamin B-6 status and elevated pyridoxal kinase levels induced by theophylline therapy in humans.. J Nutr.

[24] McCormick DB, Chen H (1999). Update on interconversions of vitamin B-6 with its coenzyme.. J Nutr.

[25] Jang YM, Kim DW, Kang TC, Won MH, Baek NI (2003). Human pyridoxal phosphatase: molecular cloning, functional expression, and tissue distribution.. J Biol Chem.

[26] Clayton PT (2006). B6-responsive disorders: A model of vitamin dependency.. J Inherit Metab Dis.

[27] Safo MK, Musayev FN, di Salvo ML, Hunt S, Claude JB (2006). Crystal structure of pyridoxal kinase from the Escherichia coli pdxK gene: Implications for the classification of pyridoxal kinases.. J Bacteriol.

[28] Li MH, Kwok F, Chang WR, Lau CK, Zhang JP (2002). Crystal structure of brain pyridoxal kinase, a novel member of the ribokinase superfamily.. J Biol Chem.

[29] Li MH, Kwok F, Chang WR, Liu SQ, Lo SC (2004). Conformational changes in the reaction of pyridoxal kinase.. J Biol Chem.

[30] Cao P, Gong Y, Tang L, Leung YC, Jiang T (2006). Crystal structure of human pyridoxal kinase.. J Struct Biol.

[31] Newman JA, Das SK, Sedelnikova SE, Rice DW (2006). The crystal structure of an ADP complex of bacillus subtilis pyridoxal kinase provides evidence for the parallel emergence of enzyme activity during evolution.. J Mol Biol.

[32] Musayev FN, di Salvo ML, Ko TP, Gandhi AK, Goswami A (2007). Crystal structure of human pyridoxal kinase: Structural basis of M(+) and M(2+) activation.. Protein Sci.

[33] Apgar JL, Tarka SM, Spiller GA (1998). Methylxanthine composition and consumption patterns of cocoa and chocolate products..

[34] Tarka SM, Morrissey RB, Apgar JL, Hostetler KA, Shively CA (1991). Chronic toxicity/carcinogenicity studies of cocoa powder in rats.. Food Chem Toxicol.

[35] Tarka SM (1982). The toxicology of cocoa and methylxanthines: A review of the literature.. Crit Rev Toxicol.

[36] Brusick D, Myhr B, Galloway S, Rundell J, Jagannath DR (1986). Genotoxicity of theobromine in a series of short-term assays.. Mutat Res.

[37] Brusick D, Myhr B, Galloway S, Rundell J, Jagannath DR (1986). Genotoxicity of cocoa in a series of short-term assays.. Mutat Res.

[38] Wada K, Ishigaki S, Ueda K, Sakata M, Haga M (1985). An antivitamin B6, 4′-methoxypyridoxine, from the seed of ginkgo biloba. L Chem Pharm Bull (Tokyo)..

[39] Fiehe K, Arenz A, Drewke C, Hemscheidt T, Williamson RT (2000). Biosynthesis of 4′-O-methylpyridoxine (ginkgotoxin) from primary precursors.. J Nat Prod 63, 185–189.

[40] Kajiyama Y, Fujii K, Takeuchi H, Manabe Y (2002). Ginkgo seed poisoning.. Pediatrics.

[41] Tutor-Crespo MJ, Hermida J, Tutor JC (2004). Activation of serum aminotransferases by pyridoxal-5′ -phosphate in epileptic patients treated with anticonvulsant drugs.. Clin Biochem.

[42] Gandhi AK, Ghatge MS, Musayev FN, Sease A, Aboagye SO (2009). Kinetic and structural studies of the role of the active site residue Asp235 of human pyridoxal kinase.. Biochem Biophys Res Commun.

[43] Safo MK, Musayev FN, Hunt S, di Salvo ML, Scarsdale N (2004). Crystal structure of the PdxY protein from Escherichia coli.. J Bacteriol.

[44] Bach S, Knockaert M, Reinhardt J, Lozach O, Schmitt S (2005). Roscovitine targets, protein kinases and pyridoxal kinase.. J Biol Chem.

[45] Tang L, Li MH, Cao P, Wang F, Chang WR (2005). Crystal structure of pyridoxal kinase in complex with roscovitine and derivatives.. J Biol Chem.

[46] Winn MD, Ballard CC, Cowtan KD, Dodson EJ, Emsley P (2011). Overview of the CCP4 suite and current developments.. Acta Crystallogr.

[47] Brunger AT, Adams PD, Clore GM, DeLano WL, Gros P (1998). Crystallography & NMR system: A new software suite for macromolecular structure determination.. Acta Crystallogr.

[48] Emsley P, Lohkamp B, Scott WG, Cowtan K (2010). Features and development of Coot.. Acta Crystallogr.

